# High Prevalence of a c.5979dupA Variant in the Dysferlin Gene (DYSF) in Individuals from a Semiarid Region of Brazil

**DOI:** 10.2174/0113892029257856231013115036

**Published:** 2023-12-20

**Authors:** Isabella A. Motta, Maria L.A. Gouveia, Ana P.M. Braga, Rafael S. Andrade, Mayra F.F. Montenegro, Sandra N. Gurgel, Keila M.F. Albuquerque, Priscilla A.N.G. Souto, Flávia P.B.F. Cardoso, Joseane S. Araujo, Mirella C.L. Pinheiro, Carlos E.P. da Silva, Pamella A.S. Gurgel, David Feder, Matheus M. Perez, Glaucia L. da Veiga, Beatriz C.A. Alves, Fernando L.A. Fonseca, Alzira A.S. Carvalho

**Affiliations:** 1 Neurorehabilitation service at Lauro Wanderley University Hospital, João Pessoa, Paraíba, Brazil;; 2 Department of Pharmacology, Centro Universitário FMABC, Santo André, SP, Brazil;; 3 Clinical Analysis Laboratory, Centro Universitário FMABC, Santo André, SP, Brazil;; 4 Department of Neurosciences – Neuromuscular service, Centro Universitário FMABC, Santo André, SP, Brazil

**Keywords:** Dysferlin, limb girdle muscular dystrophy, miyoshi myopathy, distal myopathy, genetic testing, dysferlinopathy

## Abstract

**Background:**

Dysferlinopathies represent a group of limb girdle or distal muscular dystrophies with an autosomal-recessive inheritance pattern resulting from the presence of pathogenic variants in the dysferlin gene (DYSF).

**Objective:**

In this work, we describe a population from a small city in Brazil carrying the c.5979dupA pathogenic variant of DYSF responsible for limb girdle muscular dystrophy type 2R and distal muscular dystrophy.

**Methods:**

Genotyping analyses were performed by qPCR using customized probe complementary to the region with the duplication under analysis in the DYSF.

**Results:**

A total of 104 individuals were examined. c.5979dupA was identified in 48 (46.15%) individuals. Twenty-three (22%) were homozygotes, among whom 13 (56.5%) were female. A total of 91.3% (21) of homozygous individuals had a positive family history, and seven (30.4%) reported consanguineous marriages. Twenty-five (24%) individuals were heterozygous (25.8±16 years) for the same variant, among whom 15 (60%) were female. The mean CK level was 697 IU for homozygotes, 140.5 IU for heterozygotes and 176 IU for wild-type homo-zygotes. The weakness distribution pattern showed 17.3% of individuals with a proximal pattern, 13% with a distal pattern and 69.6% with a mixed pattern. Fatigue was present in 15 homozygotes and one heterozygote.

**Conclusion:**

The high prevalence of this variant in individuals from this small community can be explained by a possible founder effect associated with historical, geographical and cultural aspects.

## 1 INTRODUCTION

Dysferlinopathies represent a group of limb girdle or distal muscular dystrophies with an autosomal recessive inheritance pattern resulting from the presence of pathogenic variants in the dysferlin gene (DYSF). This gene is located on chromosome 2p13 and encodes the dysferlin protein (OMIM#603009), which is primarily expressed in skeletal muscle, within the sarcolemma [1-4]. Classically, two phenotypes are representative of dysferlinopathy: a distribution pattern involving scapular and pelvic girdle muscles, previously described as limb-girdle muscular dystrophy (LGMD2B; MIM#253601) [4] and currently reclassified as LGMD2R dysferlin-related [5], and Miyoshi Myopathy Type 1 (MMD1; MIM#254130), involving the posterior region of the legs, which can also be caused by biallelic mutations in the ANO5 gene (MMD3; MIM#613319). The involvement of the anterior compartment is known first as Nonaka's myopathy, and its locus maps to another chromosome [3, 6]. Initially cited by Miyoshi in 1967 [7] as a distal myopathy with the first symptoms in young adults, Miyoshi's myopathy type 1, as it has become known, was exhaustively described worldwide [7, 8]. Since then, hundreds of genetic variants of DYSF have been reported; however, the same genetic variant has variable expression (both within and between families) [1].

Other phenotypes that also follow autosomal recessive inheritance have been previously described, such as proximodistal presentation, a pseudometabolic pattern, isolated hyperCKemia, and the involvement of the anterior leg compartment described by Illa *et al.* [6]. However, the progression of muscular weakness in the latter and the severity of the clinical evolution differ from Nonaka's Myopathy mentioned above [1, 6].

The estimated prevalence of dysferlinopathies varies according to the region, with a high prevalence in endogamous populations, such as populations in Maghreb, Israel, Saudi Arabia, and Iran in Jewish people from Libya and from the Caucasus and in some regions of Brazil, where the subtype of LGMD2R dysferlin-related is the most common [9-12]. Following the evaluation of a proband with LGMD2R dysferlin-related dysferlinopathy in a small Brazilian city in the northeast region called São Mamede, we found a high frequency of people with a previously described pathogenic variant located on chromosome 2p13 in exon 53 of the *DYSF* gene, characterized by the presence of duplication of adenine at position 5979 (c.5979dupA, rs398123799). Considering this finding and the few reports about epidemiological studies related to limb-girdle muscular dystrophies in South America [13], we initiated an active investigation into the frequency of LGMD2R in this village.

## 2 MATERIALS AND METHODS

This transverse and observational study was approved by the Ethics and Research Committee of Lauro Wanderley University Hospital of the Federal University of Paraíba – Brazil (protocol number 2.454.649). Interviews were conducted with a sample of inhabitants (invited to participate by the health workers) with neuromuscular complaints/signs and/or first- or second-degree family members with or without symptoms. Individuals who had no symptoms, a normal CK level, and a negative family history were excluded, considering the reference CK value of <190 IU. The creatine phosphokinase (CK) level was determined in all individuals, independent of meeting the inclusion criteria, after reading and signing the consent form.

### 2.1 Demographic and Clinical Data

The researchers gathered the following data from the sample: sex, age, age at the onset of the disease, consanguinity history, family history, and duration of the disease. All individuals underwent a neurological examination conducted by a neurologist. For individuals with muscle weakness, the muscle strength was classified based on the distribution pattern as distal, proximal, or mixed. Moreover, the presence or absence of associated atypical weakness (axial and tibial anterior localization) and fatigue was documented.

### 2.2 Genotyping

Genomic DNA was isolated from peripheral blood with the use of the Illustrate™ Blood GenomicPrep Mini Spin Kit (GE Healthcare, Little Chalfont, Buckinghamshire, UK, Cat number 28-9042-64) in accordance with the manufacturer’s recommendations. The concentration and A260/ A280 ratio of the total DNA was measured by spectrophotometry with NanoDrop Lite (Thermo Fisher Scientific, Waltham, USA).

Genotyping analyses were performed in an Applied Biosystems^®^ 7500 Real-Time PCR Systems thermal cycler (Applied Biosystems, Foster City, CA, USA) containing 15 ng of DNA, 1X TaqMan^®^ Genotyping Master Mix (Applied Biosystems, Foster City, CA, cat number 4371355) and 1X customized probe and primers complementary to the region with the duplication under analysis in the DYSF gene (Thermo Fisher). The primers used in these reactions were DYSF-For 5'GGGCAAGCTGGAAATGACCTT 3' and DYSF-Rev 5'CAGCAGGCCGCTCCT 3'; the probe for the wild-type allele was VIC 5'CATGCTCACTCTCTGCTAC 3' and the probe for the mutant allele was FAM 5'TGCTCACTCTCTTGCTAC 3'. The thermal profile for genotyping consisted of an initial step of 1 minute at 60°C and 10 minutes at 95°C, followed by 40 cycles of 15 seconds at 95°C and 1 minute at 60°C, and concluded with 1 minute at 60°C.

### 2.3 Statistical Analysis

Quantitative variables are presented using measures of central tendency and dispersion. The normality of the data was assessed using the Shapiro-Wilk test and the values were considered normal when *p* < 0.05. For nonparametric values, the Kruskal-Wallis test was applied. Statistical significance was considered at *p* < 0.05. The statistical software used for analysis was Stata version 11.0.

## 3 RESULTS

A total of 165 volunteers attended the interview. Sixty-one (36.9%) were excluded because they did not meet the inclusion criteria: all of them had a normal CK level, 41 (68%) were asymptomatic and 20 (32%) presented other symptoms due to different disorders, including ataxia, amyotrophic lateral sclerosis, paresthesia, Arnold-Chiari malformation, arthralgia, lumbago, myalgia, cramps and fatigue. The median age of individuals who were excluded was 30.6±22.6 years, 38 (62.3%) were female and 23 (37.7%) were male. Individuals who were excluded had no first- or second-degree family members affected by neuromuscular disorder. From the initial 165 volunteers, 104 (63%) individuals were evaluated clinically, and genetic tests were performed as they met the inclusion criteria. Out of the 104 included volunteers, 56 (54%) were wild-type homozygotes, 25 (24%) were heterozygous carriers of the pathogenic variant, and 23 (22%) were homozygous carriers of the pathogenic variant. The age of the evaluated volunteers (n=104) varied from 3 to 73 years of age (mean age/SD: 30.8±21.6).

### 3.1 Homozygous Group

The age of onset in this group was 21.7±7 years, varying from 14 to 39 years. The duration of symptoms was 28.5±16 years, and the age of diagnosis was 49±16 years. Thirteen (56.5%) were female. All individuals (23/100%) from this group were symptomatic, and 21 (91.3%) had family members also affected; however, only seven (30.4%) reported consanguineous marriages (restricted to consanguineous parents) (Table **1**).

In relation to the muscle weakness distribution pattern, we found that 69.6% (n=16) of individuals had a mixed pattern of weakness (proximal and distal involvement), 17.3% (n=4) had proximal weakness and 13% (n=3) had a distal weakness. No statistical significance among the three weakness groups in relation to the disease duration and CK levels was observed. Regarding associated atypical weakness (axial and tibial anterior involvement), 47.8% (n=11) presented axial weakness, while 56.5% (n=13) had tibial anterior weakness, with most of them (90%) having mixed involvement. Fatigue was present in 65,2% (n=15) of individuals and predominated in the mixed group (73.3%). The duration of disease, mean and median, was 40.2 and 38.5 years in the group with proximal weakness, 23.6 and 19 in the distal group and 26,7 and 29 in the mixed group, respectively (Tables **1** and **2**).

Concerning the CK level among homozygous individuals, the mean and median were 697 IU and 143 IU, respectively, varying according to muscle weakness distribution: 2279.5 and 517.5 IU in the proximal weakness group, 2393 and 2900 IU in the distal weakness group and 2314 and 985.5 IU in the mixed weakness group, respectively (Tables **1** and **2**).

### 3.2 Heterozygous Group

This group was composed of 25 individuals aged 25.8± 16 years, of which 15 (60%) were male. The mean and median CK levels were 140.5 and 115 IU, respectively. All individuals but one had fatigue as a symptom, and two presented mildly elevated CK levels (400 and 271 IU). No other symptoms were reported. One hundred percent of individuals had at least one affected family member, and 3 (12%) reported consanguineous marriage (Table **1**).

### 3.3 Wild-type Homozygous Group

Out of the 104 included volunteers, 56 (53,8%) did not exhibit the studied variant. These individuals aged 25±21.8 years and 33 (59%) were male. The mean and median CK levels were 176 and 127 IU, respectively. Forty-eight (85.7%) individuals from this group had at least one family member affected, and only 6 (10.7%) reported consanguineous marriages (Table **1**).

## 4 DISCUSSION

Our study provides a narrative description of the characteristics of a small population carrying a pathogenic variant in the DYSF gene from São Mamede, a small city in the Northeast Region of Brazil. In 2020, the city had a population of 7,745 inhabitants, and the estimated prevalence of homozygous individuals in this study was one affected person for every 337 healthy individuals (0.3%). However, the health agents conducted an active investigation to invite individuals with neuromuscular symptoms, so the prevalence calculation may not be applicable to heterozygous volunteers as we used a convenience sample.

Community health agents play a vital role in meeting the healthcare needs of communities, improving access to essential healthcare services, and facilitating community-based healthcare initiatives. In Brazil, these health agents have been collecting data on individuals with disabilities since 1998 to contribute to the Primary Care Information System. However, this valuable database has been underutilized for scientific research and public health policy purposes. A study conducted by Lopes *et al.* revealed that the work processes employed by community health workers led to an approximate 50% underreporting compared to data collected by researchers, emphasizing the importance of conducting further reliability assessments [14].

The Northeast Region in Brazil, especially the hinterland of Paraíba, is historically a territory with high levels of inbreeding. The average percentage of married individuals in this region is 20.19±9.13%, which is higher than the average described five decades ago [15, 16]. Although our study showed that approximately 22% of homozygous people carried the pathogenic variant reported above, most individuals claimed that they did not have consanguineous parents. Additionally, the high incidence of heterozygous individuals carrying the same variant in different families (24%) may be compatible with a founder effect. Another interesting point is that many older inhabitants of São Mamede emigrated a long time ago from the neighboring state of Rio Grande do Norte, which was conquered by Portugal in 1530 and, before that, by France. These older inhabitants specifically came from a small county called Ouro Branco, only 43.9 km from São Mamede, which is currently occupied by 4,669 inhabitants in search of better living conditions and have lost contact with previous generations [17].

In addition, other factors could have contributed to the increased prevalence of this pathogenic variant in the Paraíba hinterland, such as large families, low socioeconomic and infrastructural indexes, and low accessibility to urban centers for many years after their settlement, compared to other regions of the country. These factors may also be responsible for the high frequency of this variant [16, 18-20].

Another very important aspect is related to religious-cultural beliefs. People from this region believe that a possible curse settled in the city is a bad result of copulation between a father and a daughter. Many individuals with neuromuscular complaints, even in a wheelchair, refused to participate in the study because they felt embarrassed and responsible for the previous history of their ancestors, corroborating our doubts about the real prevalence of the variant.

As an example of common ancestry, high inbreeding and genetic drift rates were clearly evidenced by a description of spastic paraplegia, optic atrophy, and neuropathy syndrome (SPOAN; MIM#609541), an autosomal-recessive neurodegenerative disease, in Serrinha dos Pintos, a small city located within the limits of Paraíba's territory. The estimated prevalence is one affected person for every 250 inhabitants and one heterozygote for every nine inhabitants [21].

More than 400 different sequence variants have been reported in the DYSF gene, revealing high allelic heterogeneity [22]. No data were found in the 1000 Genomes [23], OMIM [24], and dbSNP [25] population databases regarding the frequency of the c.5979dupA variant. However, it was reported as a pathogenic variant in ClinVar, LOVD, and HGMD, as well as in control population databases (gnomAD, ABraOM) [26-30]. This variant has been reported in 12.2% of 370 Brazilian individuals from the South Region of the country, with autosomal-recessive limb-girdle muscular dystrophy being the most frequent variant (22.2%) in homozygotes among dysferlinopathies. The literature reported eight publications about this variant from nine different origin countries from 2000 to 2019, totaling 39 individuals. The predominant phenotype was the limb-girdle pattern [1, 31-36].

São Mamede's territory was discovered in 1702 by a group led by Sergeant Major Matias Vidal de Negreiros and Lieutenants Rodrigues Cabral and Manoel Monteiro of Portuguese ancestry. However, the city was founded 200 years later [18]. The most frequent surnames found among the homozygous and heterozygous individuals carrying the c.5979dupA variant are in accordance with the history of Brazil's discovery. The names are of Portuguese origin (Medeiros, Oliveira, Morais, and Nascimento) and less frequently of Spanish origin (Araújo and Lucena) [37].

Jewish immigration to the Iberian Peninsula occurred at different times in history, initially in the period of Nebuchadnezzar (604-562 BC). This immigration continued 600 years later, with the destruction of Jerusalem ordered by Emperor Titus, and it lasted until the diaspora itself [38]. During the 13th century, after the installation of the Holy Office Tribunal in Spain, more than 120,000 Jewish people took refuge in Portugal. In 1497, after the King of Portugal (D. Manoel I) married Isabel, the daughter of the Catholic king from Spain, the Jewish people, who still lived their traditions freely in Portuguese lands, were forced to convert to Christianity and became new Christians. Therefore, the discovery of America in 1500 and the later arrival of the Holy Inquisition in 1540 were the main reasons for the arrival of new Christians at Bahia's port [38]. The new Christians represented approximately 25 to 30% of the white Brazilian population. The majority went to the states of the Brazilian Northeast Region (Pernambuco, Paraíba, and Rio Grande do Norte) [38, 39].

During the Dutch government rule in Pernambuco (1624–1654), religious freedom attracted many persecuted Jews to this state. However, after the Dutch defeat in 1654, many Jews and new Christians left the coast and went to the Paraíba hinterland. The maintenance of Jewish culture through marriage arrangements between people from the same ethnic group, and even from the same family origin, may have influenced the consanguineous relationships occurring in this region [39].

## CONCLUSION

Considering all the above, it is possible that a small founder population has given rise to a new population resulting in the loss of alleles and consequent low genetic variability, which would lead to an increased prevalence of heterozygotes and consanguineous marriages. Our narrative description, suggesting a possible founder effect associated with a high prevalence of the pathogenic variant c. 5979dupA in Paraíba’s hinterland, is considered an important point to understand the history of a small part of this huge country, particularly the Northeast Region. Understanding these various aspects favors the planning of appropriate public policies for the reality of populations with a high prevalence of genetic diseases.

## Figures and Tables

**Table 1:** Volunteers characteristics.

**Variable**	**Homozygous**	**Heterozygous**
n	23 (22%)	25 (24%)
Gender (male/female)	10/13	10/15
Family history	21 (91.3%)	25 (100%)
Consanguinity	7 (30.4%)	NA
Mean ± SD		
Age (years)	49±16 (22-73y)	25.7±16 (7-58y)
Age of onset (years)	21.6± 7	-
Mean/Median		
CK (Mean/Median) IU	697/143	140.5/115

**Abbreviations:** Legend: CK: creatine Kinase; UI: international units; NA: not available

**Table 2:** Clinical findings among homozygous individuals (n=23).

**Weakness Pattern (%)**		**Dur Dis y (Mean/Median)**	***p***	**CK (Mean/Median)**	***p***
Proximal	17.3 (4)	40.2/ 38.5	0,271	2279.5/ 517.5	0,732
Distal	13 (3)	23.6/ 19		2393/ 2900	
Mixed	69.6 (16)	26.7/ 29		2314/ 985.5	

**Abbreviations:** Legend: Dur dis: duration of disease; y: years. CK: creatine Kinase. Kruskall Wallis test.

## Data Availability

The authors confirm that the data supporting the findings of this research are available within the article.

## LIST OF ABBREVIATIONS

CK: Creatine Phosphokinase
DYSF: Dysferlin Gene
LGMD2R: Limb-girdle Muscular Dystrophy Type 2R
OMIM: Online Mendelian Inheritance in Man
